# Sexual knowledge, attitudes and activity of men conscripted into the military

**DOI:** 10.1186/1471-2458-10-577

**Published:** 2010-09-27

**Authors:** Jiankang Chao, Yenchin Lin, Michia Ma, Yanchiou Ku, Chinghong Tsai, Mingder Shi

**Affiliations:** 1 Department of Psychiatry, Yuli Veterans Hospital, HuaLien, Taiwan, R.O.C; 2 Graduate School of Human Sexuality, Shu-Te University, Kaohsiung County, Taiwan, R.O.C; 3 Department of Statistics, National Cheng Kung University, Tainan, Taiwan, R.O.C; 4 Department of Nursing, Kaohsiung General Veterans Hospital, Kaohsiung County, Taiwan, R.O.C; 5 Department of Psychiatry, Military Kaohsiung General Hospital, Kaohsiung County, Taiwan, R.O.C; 6 Department of Pathology and Laboratory Medicine, Yung-Kang Veterans Hospital, Tainan, Taiwan, R.O.C

## Abstract

**Background:**

Military conscripts may experience a change in their attitude towards sex at times when sexual urges are at their peak during their physical growth. This study examines the experience, understanding, knowledge and attitudes regarding sexual activity of the military conscripts.

**Methods:**

Data was obtained from a cross-sectional survey of 1127 young adult military conscripts, and were evaluated in Southern Taiwan from January to July 2009, their demographic data, sexual knowledge, attitudes and activities were assessed.

**Results:**

Nearly 43% of the participants had performed penetrative vaginal intercourse at least once; 34% of the participants performed heterosexual oral sex at least once; almost 7% of participants had had homosexual intercourse, and 7.5% of participants had experienced homosexual oral sex in the past year. The mean sexual knowledge score based on 30 questions was 23.2 ± 4.0. The higher the educational level of the participants, the greater sexual knowledge they had obtained.

**Conclusion:**

This study found that 43% of unmarried young recruits had experienced premarital sexual activity. However, their sexual knowledge was insufficient and should be strengthened by sex education from an earlier age. College aged and adult learners also have sex education needs, especially with regard to integrating sexuality and life, being able to relate responsibly as sexual beings to others, the use of contraception, and about sexually transmitted disease.

**Keywords:**

Young recruits, Sexual behavior, Sexual knowledge, Sex education

## Background

In Taiwan, a significant number of youths were not aware of facts about HIV and AIDS transmission, but obtaining accurate HIV and AIDS information is an important step in preventing the spread of this epidemic. Ideally, the fight against AIDS should involve the family, education system, mass media, and society at large. However, the lack of necessary knowledge, values, and skills often results in ineffective and inconsistent HIV and AIDS prevention programs [1]. Adolescents and youths are at serious risk of contracting HIV, which accounts for half of new HIV infections in China every year [2]. In Taiwan, a survey conducted in 2000 found that 43% of boys and 25% of girls said yes to premarital sex, a significant increase from 34% and 13%, respectively, since 1995. The survey also found that the average age of the first sexual experience for boys had decreased to 16.1 years old [3]. More and more surveys now indicate that the attitude of teens towards sex has gradually become more open, and it is more acceptable for teens to engage in premarital sex.

Unwanted pregnancies may lead to increased abortions, unwanted childbearing, or early and unwanted marriage [4]. These events, in turn, may have adverse consequences on health as well as social, economic, and psychological welfare later in the life of the couple engaging in sex, as well as their children. In the past decade, young people have tended to engage in sexual activity at a younger age [5,6]. Maticka-Tydale [7] found that youth living in poverty, in rural areas and aboriginal youth carry the greatest burdens of poor sexual health. These youth groups are also the most poorly served by sex education and sexual healthcare. Taiwan is a developing country with somewhat conservative, but fast changing social, cultural, and moral norms. Attitudes towards sex have been changing rapidly, and premarital sex has been accepted by many young people [8-10]. A previous study demonstrated that male students, in comparison with their female counterparts, have had a higher level of agreement regarding premarital intercourse, and in the use of pressure and force experiencing sexual activity [11]. As the body matures, it triggers the desire to understand the concept of sexual behavior. The need to understand sex is the key determination of one's sexual behavior in later years. Bassani et al. [12] point out that child sex abuse takes place at a young age and is associated with physical violence, making serious health and developmental consequences more likely. So a correct and healthy sexual consciousness can have a constructive effect on one's growth, development, work and study, whereas the wrong sexual consciousness may negatively affect one's growth, development, work and study, and could even become a leading cause of sexual crime [13-15].

Sexual attitudes are one's subjective or objective views concerning sex. Sexual attitudes are also the notions and thoughts related to sexual knowledge, as well as ones point of view, judgment, understanding, and action taken concerning sex. Duyan and Duyan [16] suggest that whether an individual has engaged in vaginal intercourse or not is associated with a range of sexual attitudes. They also point out that religion correlates positively with more conservative attitudes towards sexuality and avoidance of certain sexual practices such as premarital and homosexual sex [16,17]. Sex education in Taiwan starts at age 10, when children are in the fifth-grade of elementary school, and ends roughly at age 19 when they are supposed to take responsibility for their own decisions [4]. However, the fact that learners have finished high school does not mean that they have no further need for sex education. College programs must do more than inform students about sex [18]. They need to help students integrate sexuality and life, relate responsibly as sexual beings to others, and help balance freedom and human values.

Under the current Military Service Act, all Taiwan male citizens between the ages of 19 and 36 (24 for a bachelors degree, 27 for a masters degree, and 30 for a doctoral degree) are considered to be of "draft age" and are subject to conscription, including those possessing dual citizenship, no matter whether they are married or not. Men conscripted into the military may experience a change of view towards sex at this time in their lives, when their sexual urges are at their strongest and they are at the peak of their growth. Men conscripted into military service have normally graduated from university or are young adults who have entered into society. Finishing university level education or having entered society does not represent having an understanding of basic sexual knowledge. Our study was aimed at providing the evidence needed to add a complete structured sex education program for the general population beyond high school student age. We have also examined the sexual knowledge, attitudes and behaviors of young men conscripted into the military. We also aimed to determine if there were differences in socio-demographic characteristics between men who did, or did not have sexual experience.

## Methods

### Subjects

Subjects selected for this study were men conscripted into the military between the ages of 18-29 in Southern Taiwan. Participants were eligible if they could read and complete the questionnaire. A psychiatric doctor and relevant staff member of the psychiatric department examined the participants individually. Subjects were excluded if they were physically unfit for military service or if they could not, or were not willing, to complete the survey. A total of 1230 participants were evaluated at Yung-Kang Veterans Hospital from January to July 2009, with 1127 (response rate = 91.6%) completed questionnaires being returned. Subjects excluded because they were unwilling to complete the survey were 46 in number; those unfit for military service numbered 23; 2 were married (this was decided later as the sample was too small); and where the survey was incomplete, i.e. in 32 cases.

### Ethical issues and Procedure

The study protocol was reviewed and approved by the Yuli Veterans General Hospital of Institutional Review Board (IRB). Before each interview, the interviewer explained the objectives and methodology of the study to each participant. The interviewer signed the survey document when consent was obtained from the participant. The staff distributed the questionnaire and informed the participants that completion of the questionnaire was voluntary.

The participants were interviewed individually by the staff members in a quiet room. Data were collected by self-reported questionnaires and in-person interviews. Participants were asked to complete the questionnaire and offered a presentation on terms used to describe sexual behavior. A covering letter accompanying the questionnaire described the purpose of the study and stressed that the information given would be kept confidential. A research assistant and a doctor were available to answer questions. After they completed the questionnaire, the participants were asked to place it in the envelope provided. Most interviews took place at Yung-Kang Veterans Hospital.

#### 1. Demographic questionnaire and sexual behavior scale

The questionnaire included 32 questions, which were mainly multiple-choice. There were 16 demographic (included the participants' height, weight, age, educational level, religion, income, and faculties) and descriptive questions (including father's educational level, mother's educational level, sex courses studied, and so on). Data was gathered using self-reported questionnaires and in-person interviews. Data on demographics, and height and weight for the body mass index (BMI), were taken by the interviewer. The BMI is a statistical measurement of the weight scaled according to height, and is defined as the body weight divided by the square of their height. The Taiwanese obesity definition was used in this study. Overweight was defined as BMI24 kg/m^2 ^and obesity was defined as BMI27 kg/m^2 ^[19,20].

The remaining questions, the (SActS), asked about social and physical factors, and frequency of sexual activity. Frequency of sexual activity was defined as never, once, 2 to 5 times, and over 5 times (including frequency of dating, embracing, kissing, caressing of chest and breast, masturbation, oral sex and sexual intercourse in homosexual or heterosexual relationships) during the last 12 months. The SActS was originally proposed by Lin [21]. The predicted Cronbach's α value of the sexual activity scale is 0.9552.

#### **2. Sexual Knowledge Scale and Sexual Attitudes Scale**

The SKS and SAS proposed by Lin [21,22], were used to construct the sexual knowledge and attitudes questionnaires. The predicted Cronbach's α value of the sexual knowledge scale was 0.804, and the corresponding value for the sexual attitudes scale was 0.714.

#### 3. Statistical Approach

Data were analyzed using the Statistical Package for the Social Sciences (SPSS), Version 14.0. The descriptive statistics for the demographic characteristics were calculated, and included frequency distributions for categorical demographic variables or type of sexual activity variables, and the means and standard deviations for a participants' response regarding their sexual attitudes and knowledge. An additional, two sample t-tests and an analysis of variance (ANOVA) were used to test the association between the age of participants, BMI, educational level, religion, educational level of parent and the responses of the participants. If the data were not in a normal distribution or not the same with consistent variance, the non-parametric Mann-Whitney U test (M-W U test) or the Kruskal-Wallis one-way analysis of variance ranks (K-W H test) was used. The Chi-square test was used to find the association between religion, participant's and their parent's educational level and sexual intercourse. The significance level was set to be P < 0.05.

## Results

### Subject characteristics

The age range of the subjects was 18-29 years, and the average age was 20.1 ± 2.1 years; the average educational level was 13.2 ± 2.6 years. Only one participant had not received education, 7 participants had an education level under primary school age, and 551 (49%) of participants had an education level above senior high school graduate level (>12 years). With respect to the educational level of parents of the participants, only 140 (12.4%) of fathers and 70 (6.2%) of mothers had an education level above senior high school graduate level respectively. Approximately 56% of subjects had a religious belief, and 81% of subjects had an income below 20,000 NT dollars. About 49% of the participants belonged to the technical science faculties in the school's major subjects. More than 63% of participants had not taken any courses related to sex, except for the required military training and nursing courses in school. The highest level of sexual intercourse was associated with income, faculties and the sex courses studied. Participants whose income fell below 20,000 NT dollars, who studied technical science, and did not take any sex courses in school, had the lowest level of sexual intercourse (Table 1).

**Table 1:** Socio-demographic characteristics of the study group in relation to having had sexual intercourse at least once during the previous 12 months.

			Sexual intercourse				
Characteristic	N	%	None		Have		**P value **^**a**^
**Education**			N	%	N	%	
≦12 years	578	51.3	337	58.3	241	41.7	**0.533**
Over 12 years	549	48.7	310	56.5	239	43.5	
**Religion**							
Yes	633	56.2	349	55.1	284	44.9	**0.08**
No	494	43.8	298	60.3	196	39.7	
**Income**							
≦20000	915	81.2	552	60.3	363	39.7	<0.001*
21001-30000	184	16.3	80	43.5	104	56.5	
30001-40000	16	1.4	9	56.3	7	43.8	
0ver 40001	12	1.1	6	50.0	6	50.0	
**Faculties**							
Technical Science	551	48.9	345	62.6	206	37.4	0.005*
Commercial Science	232	20.6	123	53.0	109	47.0	
Health Science	28	2.5	12	42.9	16	57.1	
Others	316	28	167	52.8	149	47.2	
**Father's educational level**							
No education	19	1.7	10	52.6	9	47.4	**0.584**
Primary school	238	21.1	142	59.7	96	40.3	
Junior high school	367	32.6	214	58.3	153	41.7	
Senior high school	363	32.2	209	57.6	154	42.4	
University	140	12.4	72	51.4	68	48.6	
**Mother's educational level**							
No education	25	2.2	10	40.0	15	60.0	**0.303**
Primary school	272	24.1	150	55.1	122	44.9	
Junior high school	400	35.5	235	58.8	165	41.3	
Senior high school	360	31.9	214	59.4	146	40.6	
University	70	6.2	38	54.3	32	45.7	
**Study sexual courses**							
Yes	416	36.9	223	53.6	193	46.4	0.0.48*
No	711	63.1	424	59.6	287	40.4	

a: using chi-square test; *: P value < 0.05.

### Sexual activity and demographic variables

Nearly 34% of participants had carried out heterosexual oral sex at least once in the last year, and nearly 43% of participants had had penetrative vaginal intercourse at least once. About 7.5% of participants had performed homosexual oral sex, and 7.0% had had established homosexual intercourse. Over 49% of participants had had their chest stroked by the opposite sex outside or inside their clothes, and >45% had had their genital organs stroked by the opposite sex outside or inside their clothes (see Table 2).

**Table 2:** Sexual activity during the previous 12 months (n = 1,127)

	Never		Once		2 ~5 times		Over 5 times	
	N	%	N	%	N	%	N	%
1. Dating more than one person	652	57.9	103	9.1	185	16.4	187	16.6
2. Dating one person	539	47.8	99	8.8	140	12.4	348	30.9
3. Steady dating relationship	401	35.6	303	26.9	219	19.4	204	18.1
4. Tender kiss	385	34.2	153	13.6	131	11.6	458	40.6
5. Embrace and kiss	436	38.7	106	9.4	116	10.3	469	41.6
6. Deep kissing	436	38.7	113	10.0	121	10.7	457	40.6
7. Chest stroked by the opposite sex outside the clothes	549	48.7	112	9.9	147	13.0	319	28.3
8. Chest stroked by the opposite sex inside the clothes	571	50.7	106	9.4	134	11.9	316	28.0
9. Opposite sex's genital organs stroked outside the clothes	613	54.4	115	10.2	122	10.8	277	24.6
10. Opposite sex stroked genital organs outside the clothes	612	54.3	118	10.5	119	10.6	278	24.7
11. Opposite sex's genital organs stroked inside the clothes	610	54.1	115	10.2	115	10.2	287	25.5
12. Opposite sex stroked genital organs inside the clothes	615	54.6	117	10.4	115	10.2	280	24.8
13. Oral sex with the opposite sex.	746	66.2	107	9.5	82	7.3	192	17.0
14. Sexual intercourse with the opposite sex	647	57.4	81	7.2	115	10.2	284	25.2
15. Oral sex with the same sex	1042	92.5	32	2.8	20	1.8	33	2.9
16. Sexual intercourse with the same sex	1048	93.0	22	2.0	21	1.9	36	3.2

### Sexual attitudes

The sexual attitudes questionnaire included 10 questions, scored from 1 to 4. In Table 3, the highest mean score of 3.28 indicated that most of the participants agreed that sex education can cultivate an attitude of sexual respect and responsibility. The lowest mean score of 1.72 indicated that most of the participants did not accept prostitution, even if the price was very reasonable.

**Table 3:** The descriptive statistics of sexual attitudes

Sexual attitudes items	mean	SD
Sexual attitudes ( scale 1~4)		
1. Sex education in school will not cause excessive sexuality.	2.08	0.73
2. Pre-marital sex is permitted if young couples are very close.	2.30	0.76
3. Contraceptive knowledge will not result in the pre-marital sexual behavior of youth.	2.78	0.73
4. Parents should not prevent their children from masturbating.	2.18	0.79
5. Close behavior in public may be permitted.	2.45	0.80
6. I can accept trying virtual sex.	2.19	0.77
7. I can accept one having more than two sexual partners at the same time.	1.88	0.81
8. If the price is fair, I can accept prostitution.	1.72	0.76
9. Sex education can cultivate sexual attitudes of respect and responsibility.	3.28	0.72
10. Pre-marital sex is helpful in adapting to future marriage life.	2.85	0.79

SD = standard deviation

Sexual attitude scale: 1 as totally disagree, 2 as disagree, 3 as agree, 4 as totally agree.

### Sexual knowledge

Sexual knowledge testing involved 30 questions, scored by giving one point for every correct answer per question; the results are shown in Table 4. Only 4 (~0.4%) of the 1127 participants gave correct answers to all 30 questions. Participants scoring 26 points accounted for 13.5% of the sample; only one person had the lowest score of 10 points, and the sexual knowledge mean score of 23.2 ± 4.0 was slightly low. Most of the participants believed oral contraceptives could be taken before every instance of sexual intercourse; this question only had a 34% correct answer rate. Also, 59.3% of participants believed that masturbation is harmful. In the safe period-related questions, the correct answer rate was also low. On the item regarding the fetus's gender being decided by the sperm, the correct answer rate was only 60.7%, and the correct answer rate for other reproduction-related knowledge was <70%. About 29% of the participants believed sperm was produced by the penis, and 27.4% of the participants did not know that women in the post-menstrual cycle still have a reproductive capability. Furthermore, 32.4% of the participants thought that, as long as the female menstrual cycle was irregular, there was only a minute possibility of pregnancy. The composite measure of knowledge of pregnancy prevention showed young adults in the forthcoming recruitment group had an even lower level of detailed knowledge about pregnancy prevention than they did about HIV. Evidence from this study showed that although awareness of HIV was very high among young adults, in-depth knowledge about HIV transmission and prevention was very low. For example, 20.6% of the participants believed speaking with; shaking hands with and hugging an HIV carrier are possible transmission methods (Table 4).

**Table 4:** Percentage of young adults with correct sexual knowledge

Sexual Knowledge Descriptive	%
**Knowledge of reproduction**	
1. Fetus's gender is decided by the sperm.	60.7
2. Pregnancy is impossible if the sperm do not enter the vagina.	65.5
3. Women in the post-menstrual cycle have reproductive capability.	72.6
4. Men less than 16 years old still have reproductive capability.	68.0
5. Women in the early period of pregnancy have no menstruation.	83.4
6. Sperm is not produced by the penis.	71.3
7. Whether pregnancy is achieved or not is related to the wife and husband.	92.5
8. Pregnancy is possible if intercourse does not reach a climax.	90.9
10. Reproductive ability is unrelated to the size of the penis.	82.3
**Knowledge of contraception**	
1. Oral contraceptives should not be taken only before every sexual intercourse.	86.0
2. The condom is used to prevent sperm from entering the vagina.	34.0
3. All contraceptive methods are not 100% effective.	96.3
4. Sperm can survive in the vagina about 2~3 days.	76.1
5. We can buy oral contraceptives and condoms in the drug store.	92.1
6. Pregnancy is possible if intercourse occurs only once.	82.4
7. Pregnancy is possible if the female menstrual cycle is irregular,	67.6
8. Pregnancy is possible if intercourse occurs during the two weeks before the MC.	50.3
**Knowledge of sexual health**	
1. Women should take a shower during their menstruation period.	71.4
2. Masturbation is not harmful.	40.7
3. Sperm cannot be transformed into blood and is not a source of power.	74.9
4. Nocturnal emissions will not affect health and sexual ability.	81.8
5. Consanguineous marriage will lead to a high possibility of a defective fetus.	81.6
6. The probability of pregnancy for women with normal sexual intercourse decreases with age, especially after 35 years old.	85.4
**Knowledge of AIDS**	
1. AIDS may be transmitted to anyone, not only among homosexuals.	90.1
2. Mothers with HIV will transmit AIDS to the fetus.	89.4
3. AIDS will be transmitted by sharing needles.	94.1
4. AIDS is not transmitted by speaking with, shaking hands with, hugging an HIV carrier.	79.4
5. The probability of transmitting AIDS can be reduced by using a condom.	92.3
6. HIV carriers look like normal people.	86.3

Mean score: 23.2 ± 4.0

### Subject characteristics and sexual activity, attitudes and knowledge

The religion, participant's educational level, father's educational level, mother's educational level, faculties and sex courses studied did not show a normal distribution. The Mann-Whitney and K-W H test statistics showed that participants, whether they had religious beliefs or not, did not associate with sexual attitudes and sexual knowledge (Table 5). The income of participants also had no relation and was not recorded in the Table 5. In particular, a participant with an education over high school level had significantly more sexual knowledge and more open sexual attitudes than those with an education of less than high school level. The educational level of the parents of participants, the participants' faculties and sex courses studied in school did associate with sexual knowledge. The sexual knowledge of the participants whose father had a university education level was higher than those whose father had less than a senior high school education level. Table 5 also shows participants whose mothers received an education had a higher level of sexual knowledge than those mothers not receiving an education. Because the sexual attitudes and knowledge of the participants whose parents had primary school, junior high school and senior high school education level were not significantly different, they were combined. The higher the educational level of the parents, the better their sexual knowledge, but the sexual attitudes and sexual activity of the participants were unrelated to the level of education of the parents. Those participants who studied health science and took any sex courses in school had significantly more sexual knowledge than other participants.

**Table 5:** Association of socio-demographic characteristics and sexual attitudes, sexual knowledge

	Sexual attitudes total score Mean ± SD (median)	Sexual knowledge total score Mean ± SD (median)
**Religion **^a^		
Yes (N = 633)	23.68 ± 3.35 (24.0)	23.21 ± 3.86 (24.0)
No (N = 494)	23.74 ± 3.64 (24.0)	23.05 ± 4.20 (24.0)
P value	**0.785**	**0.910**
**Participant's educational level **^a^		
≦12 years (N = 576)	23.95 ± 4.00 (24.0)	21.38 ± 4.14 (23.0)
>12 years (N = 551)	23.45 ± 2.80 (23.0)	25.00 ± 2.88 (26.0)
P value	<0.001^*^	<0.001^*^
**Father's educational level **^b^		
No education (N = 19)	24.63 ± 3.45(25.0)	19.21 ± 4.42 (20.0)
Primary & high school (N = 968)	23.68 ± 3.56 (24.0)	22.99 ± 4.00 (24.0)
University (N = 140)	23.66 ± 2.92 (24.0)	24.83 ± 3.32 (26.0)
P value	**0.154**	<0.001 ^*^
Post Hoc		NE < PHS < U
**Mother's educational level **^b^		
No education (N = 25)	23.84 ± 3.68(25.0)	20.64 ± 4.72(21.0)
Primary & high school (N = 1032)	23.70 ± 3.47 (24.0)	23.12 ± 3.97 (24.0)
University (N = 70)	23.80 ± 3.53 (23.5)	24.41 ± 3.93 (26.0)
P value	**0.703**	<0.001^*^
Post Hoc		NE < PHS < U
**Faculties **^b^		
Technical science (N = 551)	23.76 ± 3.58 (24.0)	23.02 ± 3.87 (24.0)
Commercial science (N = 232)	23.60 ± 3.16 (24.0)	23.95 ± 3.68(25.0)
Health science (N = 28)	23.21 ± 2.13(23.0)	25.32 ± 3.35 (26.0)
Others (N = 316)	23.74 ± 3.62 (24.0)	22.58 ± 4.38 (24.0)
P value	**0.542**	<0.001 ^*^
Post Hoc		T, O < C < H
**Study sexual courses **^a^		
Yes (N = 416)	23.95 ± 3.21 (24.0)	23.45 ± 3.95 (25.0)
No (N = 711)	23.57 ± 3.62 (24.0)	22.97 ± 4.04 (24.0)
P value	**0.244**	0.027 ^*^

NE = No education, PHS = Primary school, Junior high school, senior high school, U = University T = Technical science; C = Commercial science; H = Health science; O = Others

Religious belief include any participants who are pertaining to a religion, which include Christian, Buddhism, local religion, Islam, Catholic and other any participants who do not have any religion are nonreligious.

a: using Mann-Whitney U test; b: using K-W H test *: P value < 0.05.

### Factors associated with sexual intercourse

With regard to sexual intercourse, whether the participant had engaged in sexual intercourse was significantly correlated with sexual knowledge, sexual attitude (Table 6), income, faculties and any sex courses studied (Table 1), but it was not significantly correlated with age, BMI, religion or participant' and parents' educational level. The sexual knowledge scores and sexual attitude of participants with previous sexual experience were significantly higher than those without previous experience.

**Table 6:** Association of sexual intercourse and sexual attitudes, sexual knowledge, body mass index and demographic data

	intercourse		
	None(N = 647)	Have(N = 480)	P value
Sexual attitudes	24.66 ± 3.51	25.82 ± 3.25	<0.001^*^
Sexual knowledge	22.83 ± 4.06	23.69 ± 3.91	<0.001^*^
BMI	23.13 ± 5.28	23.34 ± 10.00	**0.655**
Age	20.00 ± 2.04	20.26 ± 2.18	**0.061**
Education (years)	13.33 ± 2.40	13.12 ± 2.86	**0.478**

a: using Mann-Whitney test; *: P value < 0.05.

## Discussion

The average educational level in this study was 13.2 ± 2.6 years, showing that the education level of participants was significantly higher than the past generation in Taiwan [23]. In our study we found that 43.4% of men obtained a university qualification, where as the article in comparison from 1993 they only had freshmen [23]. Most of the men conscripted into the military had had premarital sexual behavior. Noting that nearly 43% of participants had performed penetrative vaginal intercourse at least once, this results was significantly higher to a study in Beijing in 1999, which reported only 17% of male and 12% of female university students had experienced premarital sex [24]. Rates of sexual experience reported by men conscripted into the military were higher than those reported by university students in China, a finding also higher than that report by Nishimura in Mauritius [25]. He recorded that ~31% of males and 9.7% of females reported a history of sexual intercourse. This finding was higher than the investigation by Adhikari in Nepal [26], who found that of 573 college male students, ~39% of the survey respondents reported that they had had premarital sex. This was consistent with other studies conducted in Lithuania and other countries [27,28].

Our survey also found 7% of homosexual intercourse was performed, with homosexual oral sex reaching 7.5%. This finding is higher than in a Chinese study [29], in which Congo reported nearly 3.7% of Chinese male university students experienced, at least once, same-gender sexual contact. Our results were higher than Ma's report [30], who concluded that roughly 3.4% of male and 2.9% of female university students in Chinese reported having had homosexual and/or bisexual relationships. This result demonstrates that homosexual and/or bisexual people are becoming more visible in Taiwan, and there is a growing gay liberation movement in Taiwan. Another reason is that there is no policy that prohibits homosexual or bisexual individuals from serving in the military, so subjects feel free to disclose their sexual history.

This study shows a sexual knowledge means score of 23.2 ± 4.0 which is slightly low; only 34% of the participants correctly answered all questions with regard to oral contraceptives. Concerning the time in the menstrual cycle when it is safe to have sexual intercourse, the correct answer rate was only 50.3%, which may lead to a greater number of teenage pregnancies, abortions, and other negative issues. The study also highlights the importance of sex education by showing that the higher the educational level of the parents, the better the sexual knowledge of the participants. This confirms that parents are one of the main sources of knowledge and influence, as well as school and peer groups. While much effort and many resources have been earmarked for sex education in schools, family education seems to have been ignored, although it is one of the most important parts of education.

Our findings also show the higher the level of education for men conscripted into the military, and their better sexual knowledge, but these factors was unrelated to sexual intercourse. Maybe those participants with a higher level of education are predominantly in the final year of university and came to the recruitment physical examinations for future preparation. In today's, society there is easier access to information (through the internet, MSN, face-book, etc.). Readers can get more information about sex, and thereby hence enhance their sexual knowledge. As they gain more knowledge about sex, they can also become more aware of the danger of unprotected sex. Hence their sexual intercourse rate may decrease. This study also showed those who had had sexual intercourse had a better sexual knowledge and a more open sexual attitude. However, sexual intercourse was not related to age, BMI and educational levels, or religion.

### Limitations of the study

Three limitations of this study should be noted. First, we did not use a probabilistic sampling method to select the study population. The participants were young adults who came from all over the country to study at university or work in Tainan city, and who came to Young Kang veteran's hospital for a physical examination, the appointed hospital for examinations for military recruitment. There was no information regarding the participating volunteers or if they were more sexually experienced and held more liberal attitudes towards "sex". Second, a bias introduced by under-reporting is possible as premarital sex is a sensitive issue and may be considered socially unacceptable in mainly Chinese cultural settings [31]. And third, the data may be biased because the questionnaire was self-reported, with 8.4% of participates not responding to the survey. However, an attempt was made to minimize this bias by ensuring privacy during the completion of the questionnaire and using the anonymous self-administered survey.

## Conclusion

About 43% of young adult military conscripts had experienced premarital sex, with same-sex sexual intercourse accounting for 7% of contacts. However, no matter whether homosexual or heterosexual, their knowledge was still insufficient to protect themselves. Without correct sexual knowledge, unprotected sexual behavior is more likely to lead to the spread of HIV infection, and to increased abortions, unwanted childbearing, or premature and unwanted marriage. Premarital sex makes a huge impact on the young adult both physically and mentally, and other consequences such as unexpected pregnancy, unsafe and unhygienic sex may lead to irreparable harm. Educational levels are associated with sexual knowledge and this study highlights the importance of sex education in providing young adult military conscripts with adequate, up-to-date information. This enables them to develop a healthy attitude and understanding about sex, to make wise decisions before having sex, and realize the consequences of indulging in sex. Most Chinese parents are afraid to talk about sex education in front of their children. This study suggests that sex education should start prior to recruitment.

## Abbreviations

SKS: Sexual Knowledge Scale; SAS: Sexual attitudes Scale; SActS: Sexual activity scale; BMI: Body Mass Index; HIV: Human Immunodeficiency Virus; CI: confidence interval; ANOVA: analysis of variance; ROC: Republic of China.

## Competing interests

The authors declare that they have no competing interests.

## Authors' contributions

All authors contributed the design of this research. JKC and MCM performed the statistical analysis and drafted the manuscript; YCL, JKC and YCK coordinated the study in field; CHT, JKC and MDS played a major role in the field survey. All the authors of the manuscript have read and agreed to its content.

## Pre-publication history

The pre-publication history for this paper can be accessed here:

http://www.biomedcentral.com/1471-2458/10/577/prepub
